# Construction of a competing endogenous RNA network to analyse glucose-6-phosphate dehydrogenase dysregulation in hepatocellular carcinoma

**DOI:** 10.1042/BSR20220674

**Published:** 2022-06-27

**Authors:** Pengyu Wang, Xitong Yang, Dan Liu, Yunhui Yang, Yuanyuan Zhang, Guangming Wang

**Affiliations:** 1 School of Clinical Medicine, Dali University, Dali, Yunnan Province, China; 2 Center of Genetic Testing, The First Affiliated Hospital of Dali University, Dali, Yunnan Province, China

**Keywords:** biomarkers, ceRNA, G6PD, HCC, immunomodulation, miR-122-5p

## Abstract

Hepatocellular carcinoma (HCC) is a common malignant tumour with high rates of morbidity and mortality worldwide. Therefore, it is of great significance to find new molecular markers for HCC diagnosis and treatment. *G6PD* is known to be dysregulated in a variety of tumours. In addition, the ceRNA network plays a crucial role in the occurrence and development of HCC. However, the mechanism by which the ceRNA network regulates *G6PD* in HCC remains unclear. We used TCGA-LIHC data to analyse the possibility of using *G6PD* as an independent prognostic marker. Univariate Cox proportional hazards regression, multivariate Cox proportional hazards regression, and receiver operating characteristic curve analysis were used to analyse the influence of *G6PD* overexpression on the prognosis of HCC patients. We also analysed the biological function of *G6PD*, its effect on the immune microenvironment, and drug sensitivity. Finally, we constructed a ceRNA network of lncRNAs/miR-122-5p/*G6PD* to explore the regulatory mechanism of *G6PD*. *G6PD* was highly expressed in HCC, was related to pathological stage and poor prognosis, and could be used as an independent prognostic indicator of HCC. The expression of *G6PD* was closely related to the immune microenvironment of HCC. In addition, the expression of *G6PD* in HCC could be regulated by the ceRNA network. Therefore, *G6PD* can be used as an immunotherapy target to improve the survival and prognosis of HCC patients, and the ceRNA regulatory network of *G6PD* has potential diagnostic and therapeutic value for HCC.

## Introduction

Primary liver cancer is the third leading cause of cancer-related deaths worldwide and the second leading cause of cancer-related deaths in China. The number of new liver cancer cases ranks sixth among all tumour types. Hepatocellular carcinoma (HCC) is the most common form, accounting for approximately 85% of all primary liver cancers [1]. The long-term survival rate of patients with HCC is generally low, and recent data from the National Cancer Center show that the 5-year relative survival rate for HCC in China is only 12.1% [2,3]. HCC occurrence and development involve the regulation of multiple genes and different factors; for example, chronic hepatitis B infection, aflatoxin-containing food consumption, alcohol abuse, smoking, and Type 2 diabetes might increase the risk of HCC [4]. Currently, the diagnosis of HCC mainly depends on imaging and serological indicators. Imaging techniques tend to have a higher diagnostic efficacy in advanced stages of HCC [5]. Alpha-fetoprotein is a recognised serum marker of HCC. However, it is not used as a diagnostic test owing to its inadequate sensitivity and specificity [6]. Given the high incidence, high mortality, and poor prognosis of HCC, it is urgent to further study the pathogenesis of this disease at the molecular level to identify new targets for diagnosis and treatment.

The glucose-6-phosphate dehydrogenase (*G6PD*) gene located on chromosome Xq28 encodes the G6PD protein, a rate-limiting enzyme in the pentose phosphate pathway (PPP). The main biological function of the PPP is to produce reduced nicotinamide adenine dinucleotide phosphate (NADPH) and ribo-5-phosphate. The PPP is a key metabolic pathway and plays an important role in tumour cell proliferation, invasion, and metastasis [7,8]. Research has been mostly focused on the study of diseases, such as haemolysis and jaundice, caused by deletion or mutations of *G6PD* [9,10]. However, it was recently observed that *G6PD* can regulate cell transformation, proliferation, apoptosis, and angiogenesis in tumour tissues [11–13]. Increased *G6PD* expression, at both the mRNA and protein levels, in different tumour tissues increases PPP activity and promotes tumour cell growth and survival [14–16]. Therefore, to explore the biological function of *G6PD* in HCC, we aimed to test it as a new molecular marker for HCC diagnosis and treatment.

Some long non-coding RNAs (lncRNAs), circular RNAs, pseudogenes, messenger RNAs (mRNAs), and other noncoding RNAs (ncRNAs) have microRNA (miRNA)-response elements (MREs), which regulate the expression of miRNA-target genes by competitively binding with miRNA [17]. Molecules in the regulatory network that perform this function are called ceRNAs. It has been recently found that the regulatory mechanism underlying the ceRNA network plays a crucial role in HCC occurrence and development [18]. The ceRNA network in HCC is involved in various biological processes, such as cancer cell growth, epithelial–mesenchymal transformation, invasion, and metastasis [19]. LncRNAs overexpressed in the ceRNA network are also involved in different biological processes in HCC, such as cell cycle progression, autophagy, and apoptosis [20]. MiRNAs are small and evolutionarily conserving ncRNAs that regulate gene expression by degrading mRNA or binding to the 3′-UTR region of the target mRNA to inhibit its translation [21]. Abnormal expression of miRNA can affect the growth and migration of cells and affect the occurrence of cancer.

MiR-122-5p is highly expressed in normal liver cells and not expressed or less expressed in other cell types. MiR-122-5p is specifically expressed in the liver to maintain liver homeostasis and metabolism and is also a new marker of liver injury [22,23]. MiR-122-5p has been shown to be a cancer suppressor molecule that inhibits the growth, metastasis, and infiltration of cancer cells in HCC [24,25]. In the ceRNA network, lncRNAs prevent the combination of miRNA and its target gene by binding to the target miRNA, thus upregulating the expression of the mRNA. However, the mechanism underlying *G6PD* regulation by the ceRNA network in HCC has not yet been clarified. Therefore, studying the ceRNA network centred on *G6PD* regulation could reveal the molecular mechanism underlying HCC occurrence and development.

To study the mechanism underlying the effects of *G6PD* in HCC, we analysed microarray data of human HCC samples from The Cancer Genome Atlas (TCGA) database (https://tcga-data.nci.nih.gov/tcga/) [26]. We used R to determine the expression levels of *G6PD* in tumour and normal tissues, and based on the information obtained, we analysed the correlation between *G6PD* and the prognosis of patients with HCC as well as the biological role of *G6PD*. Finally, we constructed a ceRNA network of lncRNAs/miR-122-5p/*G6PD*, providing a new strategy for elucidating HCC pathogenesis.

## Methods

### Data collection and processing

On October 7, 2021, information about 424 individuals (including 374 patients with HCC and 50 normal controls) was obtained from TCGA database, including mRNA sequencing (seq), miRNAseq, and survival data. RNA expression profiles were annotated using the Ensembl gene ID. The mean of the expression levels of duplicate genes was considered the expression level of the gene. LncRNAs, miRNAs, and mRNAs in low abundance were eliminated (low abundance was defined as the mean of gene expression being >0). The study met the research requirements of TCGA and did not require ethics approval.

### Identification of differentially expressed genes (DEGs)

We used the DESeq2 package from Bioconductor in R (Version 4.1.0) to analyse the difference between the mRNA matrix files of HCC and normal samples [27]. Genes with |log_2_ fold-change (FC)| > 1.5 and *P*-adj < 0.01 were considered differentially expressed. After the identification of differentially expressed genes (DEGs), we used the pheatmap package to visualise the expression of the DEGs, after which the ggplot2 package was used to visualise the volcano plot of the up-regulated and down-regulated genes [28]. Genes with *P*-adj < 0.01, but log_2_ FC > 1.5 or log_2_ FC < -1.5 were defined as up-regulated and down-regulated, respectively.

### Pan-cancer analysis

Pan-oncogenic transcriptome data were obtained from the University of California Santa Cruz (UCSC) database (http://xena.ucsc.edu/) for 33 different cancers [29]. The differential expression of *G6PD* in these tumours was analysed using the limma package in R. Tumour types with fewer than five normal samples in each tumour were removed, and the expression levels of *G6PD* in the remaining tumour tissues were analysed.

### Expression of G6PD in HCC

HCC data in TCGA were used to analyse the differential expression of G6PD in normal tissues and HCC tumour tissues. Furthermore, we used an immunohistochemical (IHC) database (Human Protein Atlas, HPA, https://www.proteinatlas.org/) to determine the expression level of G6PD protein in cancer and normal tissues [30]. The HPA database uses the protein analysis method of IHC from normal and tumour tissues to determine protein levels. Based on the clinical data and gene expression profile of HCC in TCGA, we analysed the relationship between the expression level of G6PD and clinic-pathological features.

### Survival analysis

Kaplan–Meier (K-M) survival analysis was used to analyse survival data from the HCC dataset [31]. The Survminer package in R was used to visualise the relationship between gene expression and overall survival (OS) of patients with HCC. The prognosis and diagnostic values of *G6PD* in HCC were evaluated by K-M survival analysis combined with receiver operating characteristic curve (ROC) analysis. The predictive value of *G6PD*, univariate Cox proportional hazards regression (UCR), and multivariate Cox proportional hazards regression (MCR) analysis were performed to understand whether *G6PD* could be used as an independent prognostic marker. Statistical significance was set at *P*<0.05.

### Analysis of the biological function of *G6PD* in HCC

Different analysed HCCs were grouped according to *G6PD* expression levels, and gene set enrichment analysis (GSEA) was conducted to determine the biological processes affected by *G6PD* and its role in HCC development [32]. The screening conditions were nominal *P*<0.05, |normalize enrichment score (NES)| > 1, and false discovery rate < 0.25. R software was then used to extract the expression levels of immunomarker genes in HCC tissues, and the correlation between *G6PD* expression and tumour immune cell infiltration was analysed. In total, 28 immunomarker genes were extracted from eight types of immune cells as follows: B cells, CD8^+^ T cells, CD4^+^ T cells, natural killer (NK) cells, M1 and M2 macrophages, neutrophils, and dendritic cells [33–35]. Finally, the correlation between *G6PD* expression and immune checkpoint molecules (*PDCD1, CD274* and *CTLA-4*) was studied to explore its relationship with immune checkpoint molecular blocking therapy.

### Building the ceRNA network

A lncRNA-miRNA-*G6PD* network was constructed for *G6PD*. The miRNA-mRNA module of the ENCORI online database (https://starbase.sysu.edu.cn) was used to identify the miRNAs combination of *G6PD* [36]. In the module, we set the conditions as follows: genome = human, target = *G6PD*, programNum ≥ 2. Next, the miRDB database (http://mirdb.org/mirdb/) is used to predict the target miRNAs that regulate G6PD [37]. Finally, the target miRNA was matched with the miRNA data in TCGA-LIHC, and the miRNA with expression level of 0 was removed. We conducted co-expression analysis (with the criteria of |cor| > 0.2 and *P*<0.001) of target miRNA and G6PD to screen out more reliable miRNA. Further, we identified the interaction of miRNA and G6PD 3′-UTR by bioinformatic analysis using Targetscan (https://www.targetscan.org/vert_72/) [38].

Next, lncRNAs bound to miRNAs were predicted by the ENCORI under the following conditions: genome = human, miRNA = miR-122-5p, target = all. We conducted co-expression analysis of target lncRNAs and miR-122-5p, lncRNAs and G6PD to screen out more reliable lncRNAs. Finally, Cytoscape software was used to construct the ceRNA network regulating the *G6PD* experssion [39].

### Drug susceptibility prediction

To evaluate the value of novel biomarkers in the clinical treatment of HCC, we used R to analyse the sensitivity of therapeutic drugs from two aspects of chemotherapy drugs and targeted inhibitors. The pRRophetic package was used to calculate the half-maximum inhibitory concentration (IC50) to predict the degree of drug response in the high *G6PD* and low *G6PD* groups [40].

## Results

### *G6PD* is overexpressed in HCC and is a potential HCC biomarker

**Figure 1:**
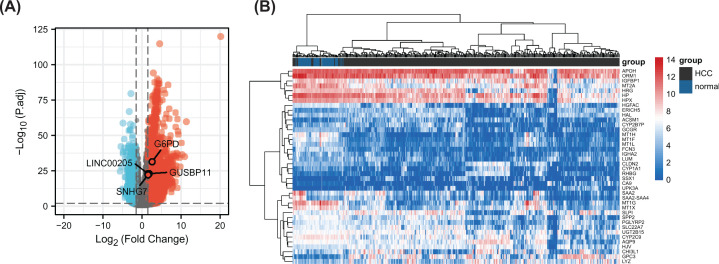
Volcano plot and heatmap maps of DEGs (**A**) Volcano plot, red for up-regulated genes, blue for down-regulated genes. (**B**) Heatmap, heatmap of the top 40 DEGs. Red and blue show differential gene expression in grouped samples. Red indicates that the expression value is high; blue indicates that the expression value is low.

To analyse *G6PD* expression levels in HCC, RNAseq data from 374 HCC tissues and 50 normal tissues were obtained from TCGA. Followed the criteria of gene |log_2_FC| > 1.5 and *P*-adj <0.01, we identified 4,800 DEGs in tumour tissues (*n*=374) when compared with the gene expression in normal tissues (*n*=50); these included 3,971 up-regulated and 829 down-regulated genes (Figure 1A). *G6PD* was identified as a DEG in HCC, with a log_2_ FC of 2.598596. The top 40 DEGs were visualised using heatmaps (Figure 1B). These observations suggested that *G6PD* overexpression is associated with HCC.

**Figure 2:**
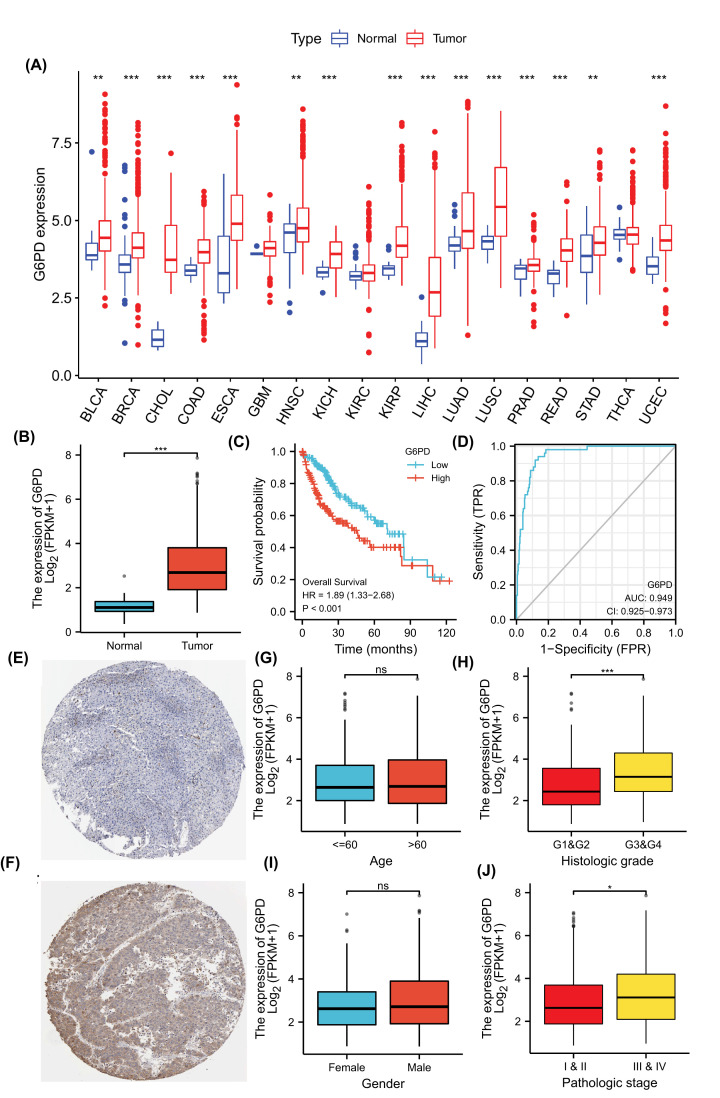
Expression and prognostic value of *G6PD* (**A**) Pan-cancer analysis, G6PD is expressed in different human tumours. (**B**) *G6PD* is expressed differently in HCC tumour tissues and normal tissues. (**C**) Kaplan–Meier survival curves were used to analyze the prognostic value of *G6PD* in HCC. (**D**) ROC analysis of the *G6PD* signatures in HCC. (**E**) Immunohistochemical images. The protein expression of G6PD in normal liver tissue was derived from HPA. (**F**) Immunohistochemical images. The protein expression of G6PD in HCC was derived from HPA. (**G–J**) The expression of G6PD in age, gender, histologic grade and pathological stage, respectively. ns: no sense; *: *P*<0.05; **: *P*<0.01; ***: *P*<0.001.

To understand the variation in *G6PD* expression in different tissue-derived cancers, we analysed *G6PD* mRNA expression levels in tissues from 33 different cancers and their respective normal tissues using RNAseq data from the UCSC database. Pan-cancer analysis showed that *G6PD* was significantly overexpressed in different tumour tissues, including HCC (Figure 2A,B).

Survival analysis showed that *G6PD* expression was correlated with the prognosis of patients with HCC, as patients with high *G6PD* expression had worse prognosis than those with low *G6PD* expression (Figure 2C). ROC analysis was used to further verify the predictive value of *G6PD* as a molecular target for HCC diagnosis and treatment. The area under the curve value for *G6PD* was 0.949 (95%CI: 0.925–0.973) (Figure 2D), indicating that the *G6PD* expression level could distinguish normal tissues from HCC tissues and can be used as a potential HCC biomarker. Furthermore, the result of ROC analysis showed that G6PD had good specificity (0.864) and sensitivity (0.940) as a marker of HCC. UCR analysis showed that pathological stage, T stage, M stage, and G6PD expression were correlated with OS. MCR analysis showed that only G6PD expression was correlated with OS. UCR and MCR analyses (Table 1) showed that the *G6PD* expression level could serve as an independent prognostic marker for patients with HCC (both *P*<0.05).

**Table 1:** Univariate and multivariate analyses of factors associated with survival of patients with HCC in TCGA database

Variable	Univariate analysis		Multivariate analysis	
	Hazard ratio (95% CI)	*P* value	Hazard ratio (95% CI)	*P* value
Age, years (≤60 vs. >60)	1.205 (0.850–1.708)	0.295	1.217 (0.757–1.956)	0.417
Gender (Woman vs. Man)	0.793 (0.557–1.130)	0.200	0.908 (0.553–1.491)	0.703
Grade (G1&G2 vs. G3&G4)	1.091 (0.761–1.564)	0.636	1.098 (0.682–1.768)	0.699
Stage (I& II vs. III& IV)	2.504 (1.727–3.631)	**<0.001**	0.188 (0.010–3.548)	0.265
T stage (T1&T2 vs. T3&T4)	2.598 (1.826–3.697)	**<0.001**	16.950 (0.925–310.459)	0.056
N stage (N0 vs. N1)	2.029 (0.497–8.281)	0.324	6.060 (0.778–47.176)	0.085
M stage (M0 vs. M1)	4.077 (1.281–12.973)	**0.017**	1.840 (0.529–6.399)	0.338
G6PD expression (Low vs. High)	1.889 (1.331–2.682)	**<0.001**	2.165 (1.335–3.510)	**0.002**

The expression of G6PD in HCC was further confirmed by immunohistochemistry, and the protein expression level of G6PD was analysed by the HPA. The results showed that G6PD was mainly expressed in the cytoplasm, and the protein expression of G6PD in HCC tissues was significantly higher than that in normal tissues (Figure 2E,F). Moreover, high expression of G6PD in HCC Li-7 cell lines was observed (Table 2).

**Table 2:** The expression level of hsa-miR-122-5p in Li-7 cell line was among the top 10 predicted targets

Target rank	Target score	miRNA name	Gene symbol	Li-7 expression
1	86	hsa-miR-122-5p	G6PD	310
2	82	hsa-miR-122-5p	PKM	305
3	56	hsa-miR-122-5p	CLIC1	294
4	51	hsa-miR-122-5p	CANX	270
5	51	hsa-miR-122-5p	NONO	142
6	69	hsa-miR-122-5p	ALDOA	116
7	85	hsa-miR-122-5p	CCNG1	80
8	97	hsa-miR-122-5p	HNRNPU	78
9	80	hsa-miR-122-5p	TUBA1C	64
10	73	hsa-miR-122-5p	LRP10	64

Notes: A predicted target with prediction score > 80 is most likely to be real. Targets with high or moderate expression are more likely to be relevant in Li-7 (High expression > 20; moderate expression 5-20; low expression 1-5).

To further determine the potential value of G6PD in clinical work, we analysed the influence of G6PD expression in TCGA-LIHC samples on clinical data. The results showed that the expression of G6PD was independent of age and gender (Figure 2G,H). The increased expression of G6PD was significantly correlated with histologic grade (Figure 2I). Moreover, in pathological stages, the expression of G6PD in stages III and IV was higher than that in stages I and II (Figure 2J). The expression of G6PD was elevated in the early pathological stage of HCC and increased with the progression of the disease.

Our analysis showed that *G6PD* is overexpressed in different cancer tissues, including HCC, and can be used as an HCC biomarker.

### *G6PD* overexpression correlates with alteration of different signalling pathways

**Figure 3:**
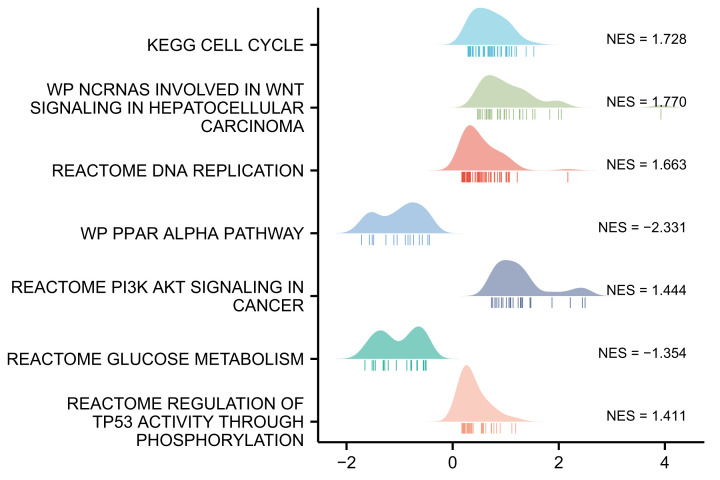
A merged enrichment plot from gene set enrichment analysis including normalised enrichment score and gene sets When G6PD was highly expressed, the significantly enriched signalling pathways were cell cycle, DNA replication, ncRNAs in WNT signalling in hepatocellular carcinoma, PI3K-AKT signalling in cancer and regulation of P53 activity through phosphorylation.

The mRNA expression matrix of patients with HCC was obtained from TCGA, and samples were grouped according to the *G6PD* expression level. GSEA was conducted using a molecular characteristic database to further understand the biological processes, signalling pathways, and roles of *G6PD* in HCC. GSEA showed that high *G6PD* mRNA expression correlated with cell cycle progression (*P*=0.001045, NES = 1.728131), DNA replication (*P*=0.003119, NES = 1.662617), and involvement of non-coding RNA in the WNT pathway in HCC (*P*=0.001087, NES = 1.7703589). Additionally, we observed a correlation between *G6PD* expression levels and the PI3K-AKT signalling pathway in cancer (*P*=0.021368, NES = 1.443601) and the regulation of P53 activity via phosphorylation (*P*=0.036756, NES = 1.411444). In contrast, low *G6PD* mRNA expression correlated with the PPARα pathway (*P*=0.004386, NES = −2.330786) and glucose metabolism (*P*=0.024390, NES = −1.353922) (Figure 3). These observations suggest an association between *G6PD* overexpression and alterations in a variety of signalling pathways.

### *G6PD* overexpression correlates with the up-regulation of immune cell markers and immune checkpoint genes in HCC

To explore the relationship between *G6PD* expression and different immune infiltrating cells, the expression levels of different immune cell markers in HCC tumours were extracted using R software and correlated with *G6PD* expression levels. B cells, CD8^+^T cells, CD4^+^T cells, NK cells, M1 macrophages, M2 macrophages, neutrophils, and dendritic cells were positively correlated with *G6PD* expression in HCC tumours (*P*<0.05, cor > 0.1; Table 3). These results suggest that *G6PD* expression in HCC is closely related to immune cells in the tumour immune microenvironment.

**Table 3:** Correlation analysis between G6PD and markers of immune cells

Immune cell	Gene	cor	*P* value
B cell	CD19	0.282931	<0.001
B cell	CD79A	0.191088	0.000201
CD8+ T cell	CD8A	0.241276	0.000003
CD8+ T cell	CD8B	0.264401	<0.001
CD4+ T cell	CD4	0.179514	0.000495
M1 macrophage	NOS2	-0.098838	0.056170
M1 macrophage	IRF5	0.342395	<0.001
M1 macrophage	PTGS2	0.132085	0.010556
M2 macrophage	CD163	0.193447	0.000172
M2 macrophage	VSIG4	0.276690	<0.001
M2 macrophage	MS4A4A	0.246326	0.000002
Neutrophil	CEACAM8	0.091118	0.078423
Neutrophil	ITGAM	0.482054	<0.001
Neutrophil	CCR7	0.099191	0.055319
Dendritic cell	HLA-DPB1	0.354284	<0.001
Dendritic cell	HLA-DQB1	0.275923	<0.001
Dendritic cell	HLA-DRA	0.318123	<0.001
Dendritic cell	HLA-DPA1	0.279320	<0.001
Dendritic cell	CD1C	0.151630	0.003287
Dendritic cell	NRP1	0.262827	<0.001
Dendritic cell	ITGAX	0.436201	<0.001
Natural killer cell	KIR2DL1	0.003966	0.939062
Natural killer cell	KIR2DL3	0.076028	0.142236
Natural killer cell	KIR2DL4	0.233855	0.000005
Natural killer cell	KIR3DL1	-0.025447	0.623740
Natural killer cell	KIR3DL2	0.130731	0.011386
Natural killer cell	KIR3DL3	0.072229	0.163319
Natural killer cell	KIR2DS4	-0.005967	0.908433

**Figure 4:**
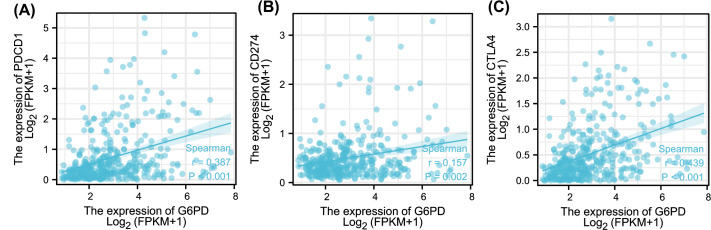
Scatter plot of correlation between G6PD and immune checkpoint (**A**) The expression level of *G6PD* was positively correlated with *PDCD1* (*r*=0.387, *P*<0.001). (**B**) The expression level of *G6PD* was positively correlated with *CD274* (*r* = 0.157, *P*=0.002). (**C**) The expression level of *G6PD* was positively correlated with *CTLA4* (*r*=0.439, *P*<0.001).

To further explore the role of *G6PD* in the tumour immune microenvironment, we also analysed the association between *G6PD* and immune checkpoint molecules (*PDCD1, CD274* and *CTLA4*). The levels of *G6PD* and PDCD1 (*r*=0.387, *P*<0.001), CD274 (*r*=0.157, *P*=0.002), and CTLA4 (*r*=0.439, *P*<0.001) were positively correlated in HCC (Figure 4), suggesting that *G6PD* in HCC affects the efficacy of immunotherapy.

### Correlation between G6PD expression level and drug sensitivity

**Figure 5:**
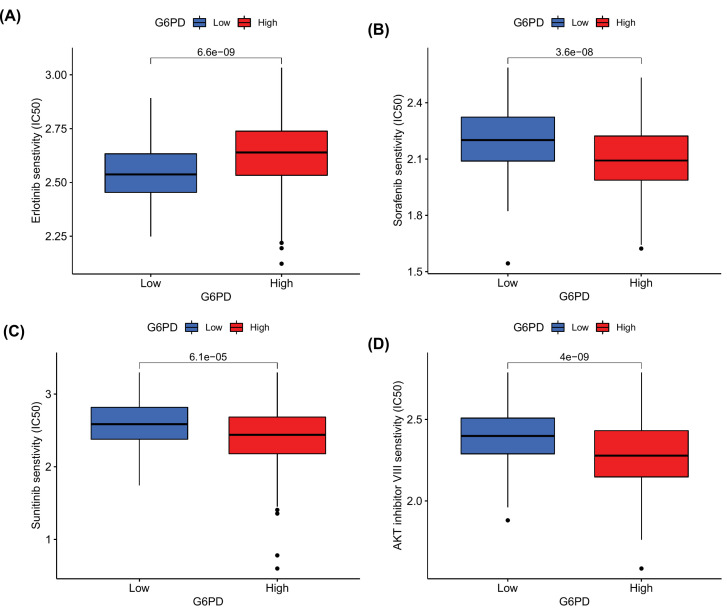
Sensitivity of drugs in different G6PD subgroups (**A**) Erlotinib (**B**) Sorafenib (**C**) Sunitinib (**D**) AKT inhibitor VIII.

We used drug sensitivity analysis to predict the sensitivity of multiple target drugs in different subgroups of populations. Interestingly, only erlotinib was more effective in the low *G6PD* population (Figure 5A). Sorafenib, sunitinib, and AKT inhibitors VIII showed better therapeutic efficacy in the HCC high *G6PD* group (Figure 5B,D). The prediction of sensitivity of these drugs can provide useful guidance for the clinical treatment of HCC patients.

### *G6PD* expression in HCC is negatively regulated by miR-122-5p

**Figure 6:**
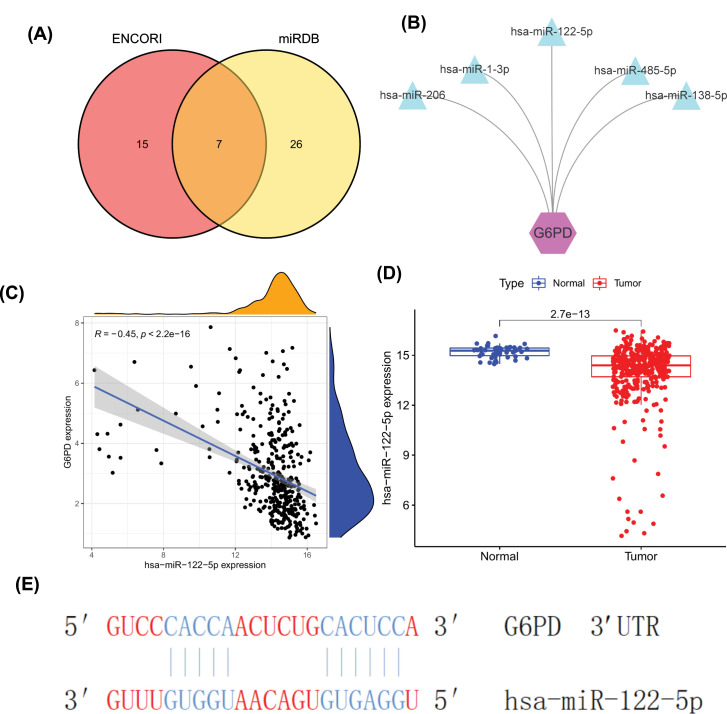
Prediction of key upstream miRNAs (**A**) Veen diagram shows overlapping miRNAs in two databases. MiRNAs regulating *G6PD* were predicted by ENCORI and miRDB databases, respectively. (**B**) Network diagram of 5 pairs of miRNAs combined with *G6PD*. (**C**) Scatter plot of correlation between miR-122-5p and *G6PD* expression (*R* = −0.45,  *P*<2.2e−16). (**D**) Difference expression of miR-122-5p between HCC and normal tissues. Low expression of miR-122-5p in HCC (*P*=2.7e-13). (**E**) Map of binding sites of miR122-5p/*G6PD*.

To clarify the regulatory mechanism underlying *G6PD* overexpression in HCC, we explored the miRNAs and lncRNAs that are implicated in the regulatory mechanism of ceRNAs. We predicted 22 miRNA-G6PD pairs using the ENCORI database and 33 pairs from the miRDB database. The intersection of the two databases was obtained using a Venn graph to obtain 7 target miRNAs (Figure 6A). Screening miRNAseq data for HCC allowed us to identify five pairs of miRNA-*G6PD* relationships (Figure 6B). Co-expression analysis results showed that only miR-122-5p was negatively correlated with *G6PD* expression (*r* = −0.45, *P*<2.2e-16; Figure 6C). Meanwhile, differential expression analysis showed that miR-122-5p was significantly down-regulated in HCC tumours (*P*=2.7e-13; Figure 6D). Finally, the binding site map of *G6PD* targeted regulation by miRNA was drawn using the Targetscan, and its binding class was 7mer-A1 (Figure 6E). In addition, high expression of *G6PD* was found in the Li-7 cell line of HCC and targeted binding to miR-122-5p was predicted (source = 86, Table 2). In summary, we predicted an interaction between *G6PD* and miR-122-5p and observed a negative correlation between their expression levels. In summary, we predicted that there was a negative regulatory relationship between *G6PD* and miR-122-5p.

### CeRNA network to control *G6PD* levels

**Figure 7:**
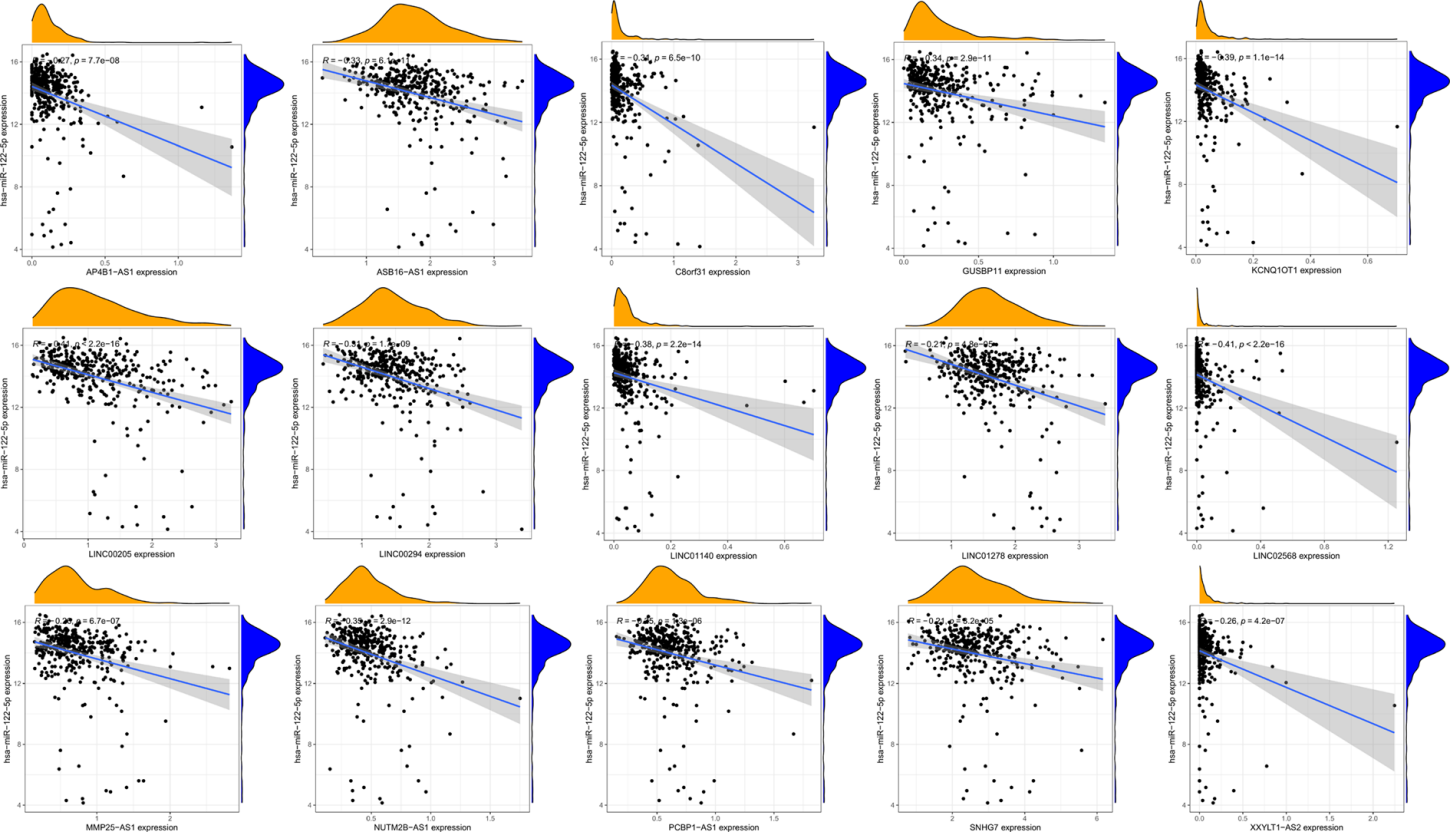
Scatter plot of correlation between 15 lncRNAs and *miR-122-5p*, respectively AP4B1-AS1, ASB16-AS1, C8orf31, GUSBP11, KCNQ1OT1, LINC00205, LINC00294, LINC01140, LINC01278, LINC02568, MMP25-AS1, NUTM2B-AS1, PCBP1-AS1, SNHG7, XXYLT1-AS2.

**Figure 8:**
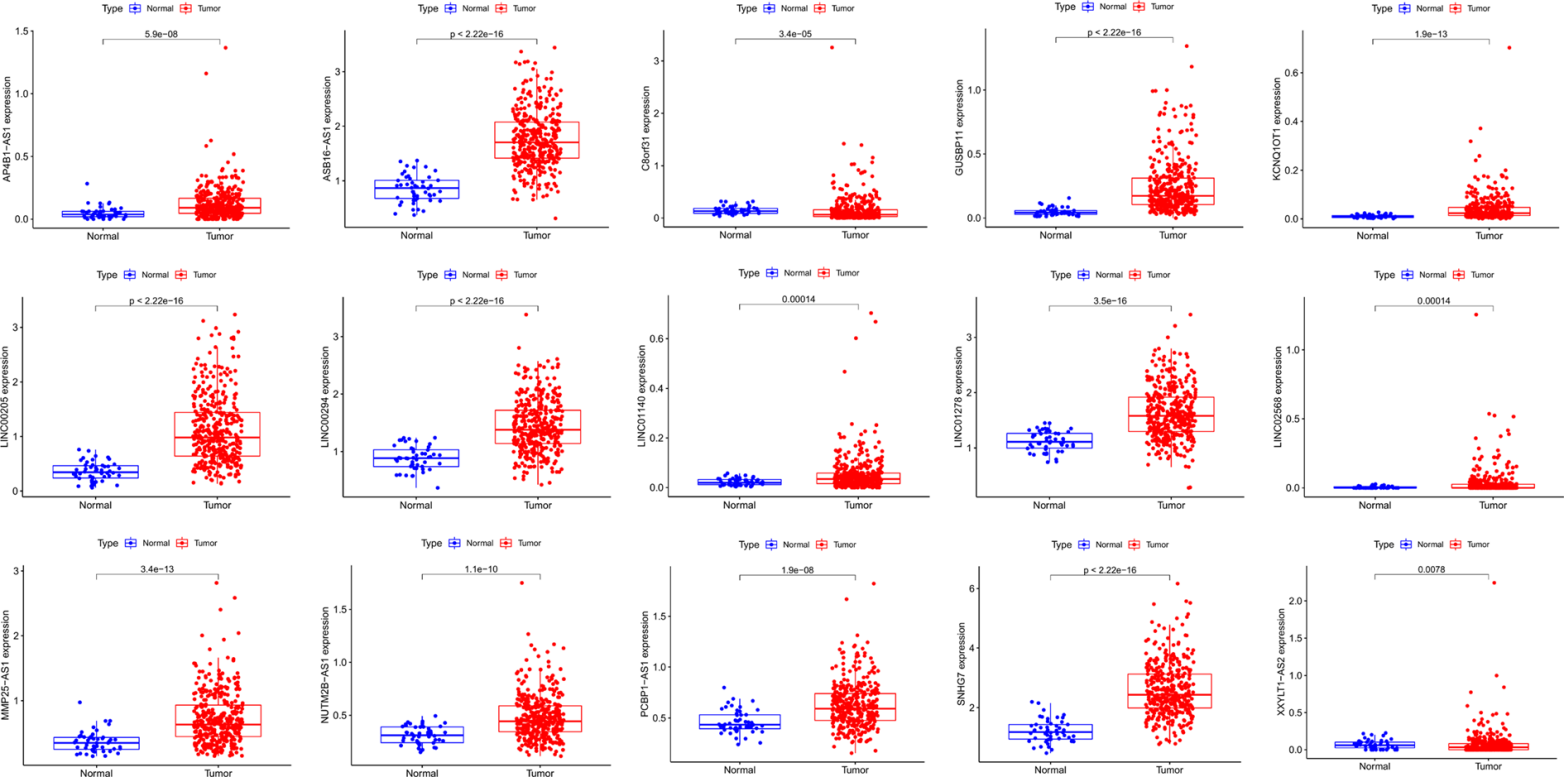
Box point composite diagram. The expression of 15 lncRNAs in HCC and adjacent normal tissues AP4B1-AS1, ASB16-AS1, C8orf31, GUSBP11, KCNQ1OT1, LINC00205, LINC00294, LINC01140, LINC01278, LINC02568, MMP25-AS1, NUTM2B-AS1, PCBP1-AS1, SNHG7, XXYLT1-AS2.

**Figure 9:**
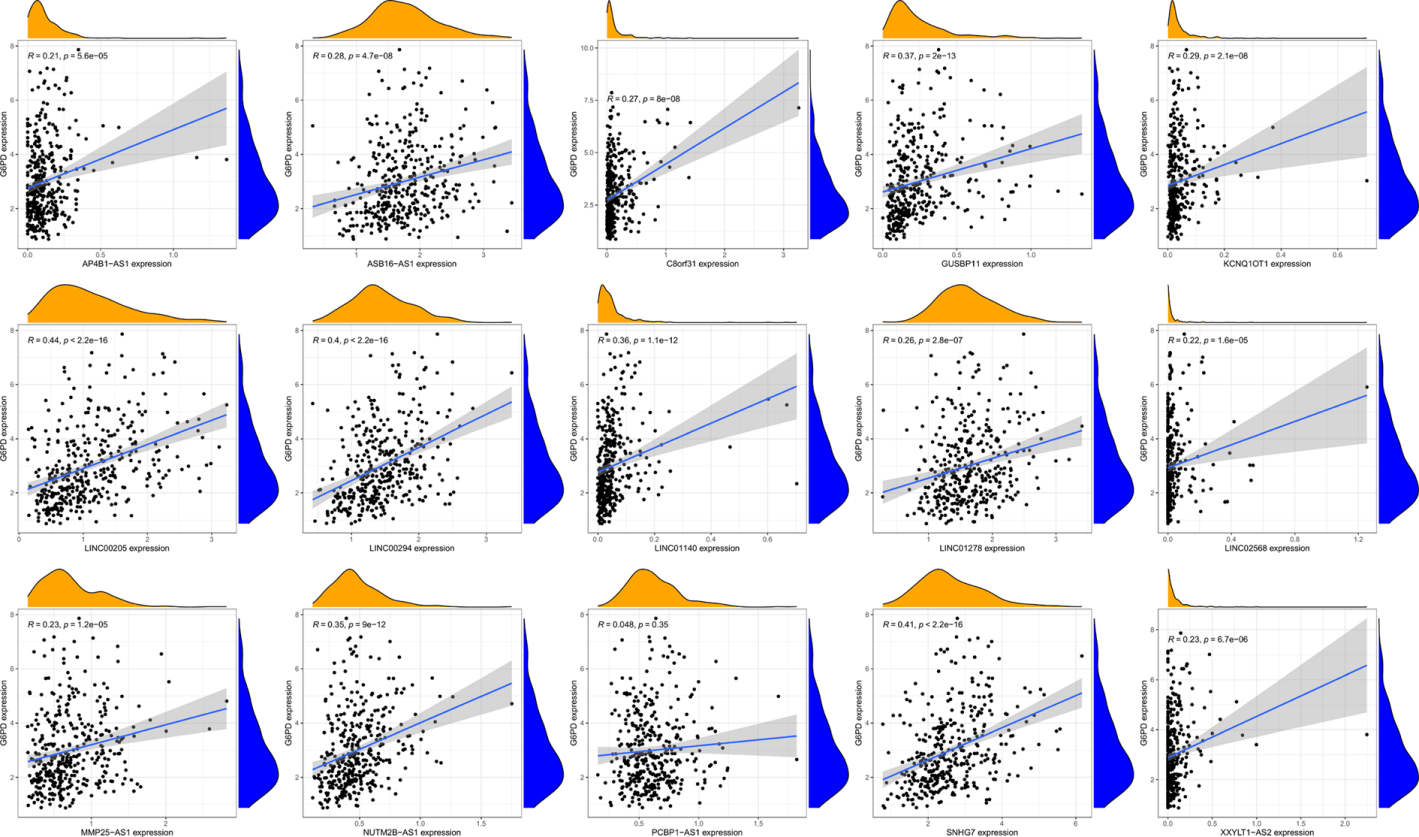
Scatter plot of correlation between 15 lncRNAs and *G6PD*, respectively AP4B1-AS1, ASB16-AS1, C8orf31, GUSBP11, KCNQ1OT1, LINC00205, LINC00294, LINC01140, LINC01278, LINC02568, MMP25-AS1, NUTM2B-AS1, PCBP1-AS1, SNHG7, XXYLT1-AS2.

To explore the effect of miR-122-5p down-regulation in HCC, the ENCORI database was used to predict lncRNAs that target miR-122-5p. In total, 101 lncRNA-miRNA pairs were identified. Next, we analysed the differential expression of 101 lncRNAs in TCGA-LIHC samples. Subsequently, we conducted a co-expression analysis of target lncRNA and miR-122-5p. We identified 15 lncRNAs with expression patterns correlated with those of miR-122-5p (Table 4) and built a scatter diagram showing these correlations (Figure 7). Additionally, we compared the expression levels of the 15 identified lncRNAs in HCC tumour tissues with those in normal tissues (Figure 8) and found that all the identified lncRNAs were highly expressed in HCC tumours. Finally, we performed a correlation analysis of the expression levels of the 15 lncRNAs and those of *G6PD* in HCC (Figure 9); expression levels of 14 lncRNAs showed a positive correlation with *G6PD* expression (Table 5).

**Table 4:** Co-expression analysis of target lncRNAs and hsa-miR-122-5p

lncRNA	miRNA	cor	pvalue
AP4B1-AS1	hsa-miR-122-5p	-0.274893	P<0.001
ASB16-AS1	hsa-miR-122-5p	-0.333474	P<0.001
C8orf31	hsa-miR-122-5p	-0.314033	P<0.001
GUSBP11	hsa-miR-122-5p	-0.338875	P<0.001
KCNQ1OT1	hsa-miR-122-5p	-0.387491	P<0.001
LINC00205	hsa-miR-122-5p	-0.411210	P<0.001
LINC00294	hsa-miR-122-5p	-0.308163	P<0.001
LINC01140	hsa-miR-122-5p	-0.383268	P<0.001
LINC01278	hsa-miR-122-5p	-0.210268	0.000048
LINC02568	hsa-miR-122-5p	-0.413327	P<0.001
MMP25-AS1	hsa-miR-122-5p	-0.255792	0.000001
NUTM2B-AS1	hsa-miR-122-5p	-0.354476	P<0.001
PCBP1-AS1	hsa-miR-122-5p	-0.249049	0.000001
SNHG7	hsa-miR-122-5p	-0.214748	0.000032
XXYLT1-AS2	hsa-miR-122-5p	-0.259494	P<0.001

**Table 5:** Co-expression analysis of target lncRNAs and G6PD

lncRNA	Gene	cor	*P* value
AP4B1-AS1	G6PD	0.207847	<0.001
ASB16-AS1	G6PD	0.280386	<0.001
C8orf31	G6PD	0.274580	<0.001
GUSBP11	G6PD	0.371584	<0.001
KCNQ1OT1	G6PD	0.287440	<0.001
LINC00205	G6PD	0.442060	<0.001
LINC00294	G6PD	0.400049	<0.001
LINC01140	G6PD	0.358563	<0.001
LINC01278	G6PD	0.264049	<0.001
LINC02568	G6PD	0.221990	<0.001
MMP25-AS1	G6PD	0.226075	<0.001
NUTM2B-AS1	G6PD	0.346909	<0.001
PCBP1-AS1	G6PD	0.048285	0.354189
SNHG7	G6PD	0.408603	<0.001
XXYLT1-AS2	G6PD	0.231693	<0.001

**Figure 10:**
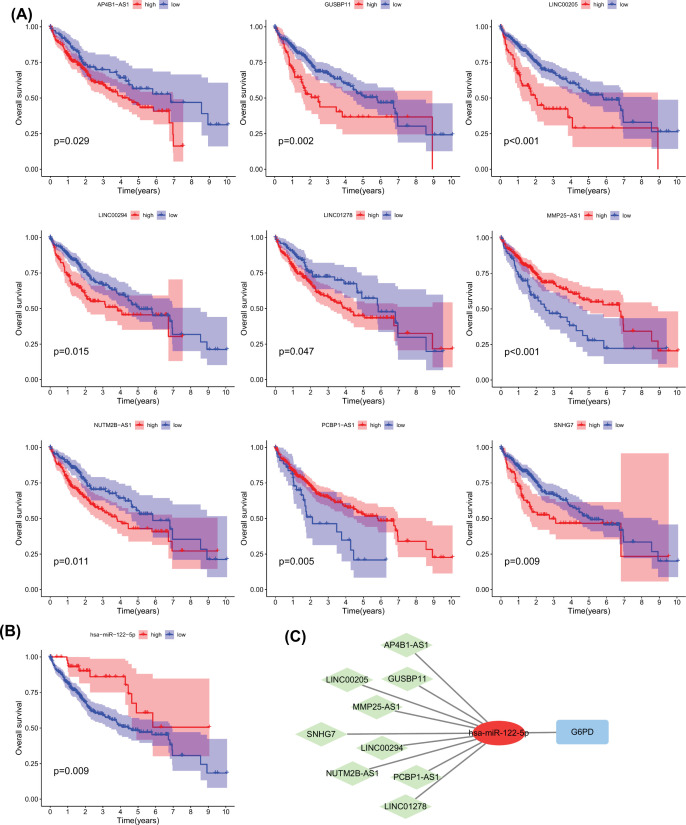
ceRNA network (**A**) Kaplan–Meier analysis for OS of HCC patients with 9 lncRNAs expression in ceRNA network. (**B**) Kaplan–Meier analysis for OS of HCC patients with *miR-122-5p* expression in ceRNA network. (**C**) CeRNA networks that regulate *G6PD* expression.

This analysis allowed us to remove the lncRNAs with large discrepancies in expression from the ceRNA network and subsequently perform a K-M survival analysis on the lncRNAs (Figure 10A). Survival analysis based on lncRNAs showed that the expression levels of nine lncRNAs were correlated with HCC survival. In addition, miR-122-5p was observed to be down-regulated in HCC, and patients with HCC with low miRNA expression had poor prognosis (Figure 10B).

These results showed that lncRNAs such as SNHG7, LINC00205, and GUSBP11 were significantly up-regulated in HCC and were associated with poor survival prognosis. Co-expression analysis showed that these lncRNAs were correlated with the expression of miR-122-5p and G6PD, indicating a potential regulatory relationship. Finally, a prognostic ceRNA network containing the nine lncRNAs and miR-122-5p was constructed based on *G6PD* expression using Cytoscape (Figure 10C). Our study suggests that G6PD expression in HCC is regulated by ceRNA, thus affecting HCC patient survival and prognosis.

## Discussion

In this study, we observed that *G6PD* expression in HCC tissues was significantly higher than that in normal tissues. Patients with HCC with high *G6PD* expression showed worse prognosis and lower survival rates than those with low expression. In addition, our analyses suggest that *G6PD* expression is an independent prognostic risk factor for HCC and that its overexpression is correlated with cell cycle progression and the involvement of non-coding RNAs in the WNT pathway in HCC. We also found a correlation between *G6PD* expression and the PI3K/AKT signalling pathway in cancer, the PPARα pathway, glycolysis, and gluconeogenesis, suggesting a correlation with tumour proliferation. *G6PD* expression was also correlated with eight types of immune cells in the tumour immune microenvironment and positively correlated with the expression of three types of immune checkpoints. These data suggest that high *G6PD* expression plays an important role in the immune response and immunotherapy of HCC. Our drug sensitivity analysis showed that HCC patients with high *G6PD* expression were more sensitive to first-line drugs, such as sorafenib and sunitinib. Finally, we constructed a ceRNA network to study the regulation of *G6PD* expression in HCC tissues.

The PPP is the most active pathway in the liver. G6PD is a key enzyme that regulates the rate of the PPP and is abnormally expressed in HCC tissues, where it activates the PPP to provide NADPH and pentose for HCC cell proliferation. Pentose is a raw material used for nucleic acid synthesis in the body, and NADPH can consume excessive reactive oxygen species in abnormally proliferating tumour cells, making tumour cells resistant to oxidative stress and promoting tumour cell survival. High *G6PD* expression influences HCC development through a variety of pathways. It was shown that *G6PD* promotes cell proliferation and participates in tumourigenesis through the reprogramming signalling pathway [41]. The WNT/β-catenin pathway is abnormally activated in HCC and is involved in tumourigenesis, drug resistance, proliferation, and metastasis of tumour cells [42]. The WNT signalling pathway promotes tumour cell proliferation and differentiation, which might be caused by the polarisation of M2 macrophages [43]. Furthermore, the WNT signalling pathway could influence HCC cell proliferation by regulating the cell cycle. The abnormal regulation of cell cycle processes leads to tumourigenesis [44]. Similarly, high *G6PD* expression is correlated with activation of the PI3K/AKT pathway. The PI3K/AKT pathway promotes the Warburg effect by increasing enzyme activity in glycolysis as well as the membrane translocation and expression of glucose transporters [45]. Activation of the PI3K/AKT pathway ensures that the energy requirements for tumour cell proliferation and differentiation are met. The present study showed that up-regulated *G6PD* in HCC is correlated with activation of the aforementioned pathways to contribute to HCC occurrence and development.

Our study also revealed that the *G6PD* expression level is correlated with that of immune markers and immune checkpoint molecules. These results suggest that *G6PD* is involved in tumour immunity and contributes to the proliferation, differentiation, metastasis, and immune evasion of HCC tumour cells. *G6PD* not only strongly correlates with immune markers of M2 macrophages but also promotes M2 macrophage polarisation through the WNT signalling pathway [43], suggesting its involvement in the regulation of tumour-associated macrophage polarisation, favouring modification of the tumour microenvironment by tumour cells to promote their proliferation. M2 macrophages play an important role in the formation of an immunosuppressive microenvironment [46]. Notably, NK cells showed dysfunction in HCC [47], but we found that G6PD was associated with KIR2DL4, a marker of NK cells. Recent studies have found that blocking the binding of KIR2DL4 to the ligand on NK cells can restore the sensitivity of breast cancer to trastuzumab [48]. This may lead to the identification of new targets for immunotherapy in HCC. Moreover,tumour cells in HCC activate immune checkpoints to inhibit the immune respone. The use of immunotherapy to block immune checkpoint molecules was shown to be effective for treating HCC [49]. In the present study, *G6PD* was strongly correlated with the expression of immune checkpoint molecules, suggesting that *G6PD* overexpression induces immune escape of tumour cells in HCC. In conclusion, *G6PD* might be involved in the immune invasion of the tumour microenvironment and in the attenuated anti-tumour response in HCC treatment.

Many patients gained no benefit from sorafenib treatment and suffered extreme adverse events and heavy financial burdens [50]. Thus, patients who are most likely to benefit from this therapy need to be identified. The results of this study may provide guidance for the clinical use of sorafenib. Considering that HCC patients with high *G6PD* levels have better sensitivity to sorafenib and the role of *G6PD* in the immune microenvironment, the combination of immune checkpoint inhibitors (CPIs) can be considered, which may yield better efficacy. Chen et al. proposed a strategy of sorafenib combined with CPIs in the treatment of HCC in a mouse model; results revealed significantly delayed tumour growth and metastasis [51]. Also, good results have also been achieved in the study of combined CPIs. The combination of atezolizumab and bevacizumab showed a better clinical significance than sorafenib in unresectable HCC patients in China [52]. Our results also indicate that HCC populations with high G6PD levels may be resistant to erlotinib; however, the specific mechanism remains to be studied.

As *G6PD* is a potential oncogene in HCC, it is necessary to study the effects of abnormal *G6PD* activation. Recent studies have found that noncoding RNAs can not only regulate *G6PD* expression via different processes but also participate in HCC occurrence and development [41]. To clarify the regulatory mechanism underlying *G6PD* overexpression in HCC, we constructed a lncRNA-miRNA-*G6PD* ceRNA network, which allowed for the identification of genes affected by abnormal expression of miRNAs [53] and lncRNAs [54]. Our observations suggest the up-regulation of *G6PD* expression mediated by the nine lncRNAs via binding to miR-122-5p. The miR-122-5p has been shown to be a tumour suppressor gene in many tumours. MiR-122-5p inhibits the proliferation and metastasis of tumour cells by inhibiting GIT1 in gastric cancer [55]. Previous studies have shown that expression of miR-122-5p is significantly reduced in HCC and can be restored after taurine treatment to inhibit glycolytic activity and ultimately metabolic activity of HCC cells to affect cancer cell proliferation, when overexpression of miR-122-5p significantly inhibited the proliferation, migration and invasion of HCC cells [56,57]. Our study revealed nine lncRNAs up-regulated in HCC and correlated with *G6PD* expression. The expression levels of these nine lncRNAs were also associated with HCC survival.

Our study showed that SNHG7 is abnormally highly expressed in HCC patients, and HCC patients with high-SNHG7 have a poor prognosis. LncRNA SNHG7 can also promote the growth and proliferation of multiple tumour cells [58]. Highly expressed SNHG7 is associated with a poor prognosis in colorectal cancer [59]. A study revealed that SNHG7 promotes the development of HCC by sponging miR-122-5p to regulate FOXK2 [24]. In our ceRNA, SNHG7 could regulate G6PD through sponge miR-122-5p in HCC; this provides novel insights regarding the SNHG7/miR-122-5p regulatory model. It has also been found that LINC00205, an emerging lncRNA, is highly up-regulated in lung cancer, gastric cancer, and retinoblastoma and is associated with a poor prognosis [60–62]. Studies have proved that LINC00205 promotes the proliferation and migration of HCC cells by targeting the expression of miR-122-5p [57]. In our study, LINC00205 is the first model to regulate G6PD expression as a ceRNA molecular sponge miR-122-5p. These novel abnormal ceRNA regulatory networks proposed in this study can provide new insights into the regulatory mechanism of G6PD and serve as a potential target for HCC immunotherapy.

In summary, we systematically studied the expression, prognostic value, and biological role of *G6PD* in HCC and confirmed that *G6PD* is an oncogene in HCC. We constructed a novel lncRNA/miR-122-5p/*G6PD* ceRNA network, revealing the potential regulatory mechanism underlying *G6PD* dysregulation. Each molecule in this novel ceRNA network has a high prognostic value for HCC. However, *in vitro* and *in vivo* experiments are needed to explore the abundance of molecules in the ceRNA network and further confirm the role of *G6PD* and its regulatory network in HCC. Our results suggest that *G6PD* can be used as an immunotherapy target to improve the survival and prognosis of patients and that the ceRNA regulatory network of *G6PD* has potential diagnostic and therapeutic value in HCC.

## Data Availability

Publicly available datasets were analyzed in this study. These data can be found here: TCGA database (https://portal.gdc.cancer.gov/) and UCSC database (http://xena.ucsc.edu/).

## Abbreviations

ceRNA: competing endogenous RNA
DEG: differentially expressed gene
FC: log_2_ fold-change
GSEA: gene set enrichment analysis
G6PD: glucose-6-phosphate dehydrogenase
HCC: hepatocellular carcinoma
HNSC: head and neck squamous cell carcinoma
HPA: Human Protein Atlas
IHC: immunohistochemical
K-M: Kaplan–Meier
LIHC: liver hepatocellular carcinoma
lncRNA: long non-coding RNA
MCR: multivariate Cox proportional hazards regression
miRNA: microRNA
mRNA: messenger RNA
NADPH: nicotinamide adenine dinucleotide phosphate
ncRNA: non-coding RNA
NES: normalize enrichment score
NK: natural killer
OS: overall survival
PPP: pentose phosphate pathway
seq: sequencing
TCGA: The Cancer Genome Atlas
UCEC: Uterine Corpus Endometrial Carcinoma
UCR: univariate Cox proportional hazards regression
UCSC: University of California Santa Cruz
